# Supplementary material for: The influence of the native lung on early outcomes and survival after single lung transplantation
Source: PLoS One. 2021 Apr 7;16(4):e0249758. doi: 10.1371/journal.pone.0249758 (PMC8026083; doi:10.1371/journal.pone.0249758)
Supplement: S2 File — (PDF) [file pone.0249758.s002.pdf]

FORM OF INFORMED CONSENT FOR THE DONATION OF ORGANS AND/OR TISSUES FOR TRANSPLANTATION

Mr./Mrs. …………………………………………………………………………., with birth date (dd/mm/yyy):…/…/…., National Identity Card nº:……………., and address:……………………………….., City:………….., postal code:………… Country:………

As next-of-kin of the deceased…………….…………………………, declares that there is no evidence of express opposition in the life of the deceased so that proceed to the extraction of………………………………………………………
……………………………………………………………………………………………
of the deceased, in order to be transplanted to patients who need them to try to save their lives or improve their health.

In the event that they are not valid for transplantation, their use for research purposes in projects approved by the corresponding Ethics and Research Committees in order to obtain knowledge that can benefit other patients is authorized.

Any observation you wish to record: ……………..………………………………….
…………………………………………………………………………………………….

Signed: …

Date: …
Hospital: …

The Coordinator Mr./Mrs. ………………………………………………………… states that after carrying out the pertinent verifications, there is no proof in life of express refusal to donate organs and/or tissues by this deceased.

Signed: …

Date: …
Hospital: …

REVOCATION

Mr./Mrs. .... National Identity Card nº .... on date (dd/mm/yyy).../.../.....
I revoke the consent given on the date (dd/mm/yyy).../.../....and I do not want the donation to proceed.

Signature of family member/legal representative......................

Signature of the professional.........................

REFUSAL OF CONSENT

I deny consent for donation.

Name of the professional reporting ....

SIGNATURE OF FAMILY MEMBER/LEGAL REPRESENTATIVE

Date (dd/mm/yyy):

SIGNATURE OF THE PROFESSIONAL REPORTING ....

Date (dd/mm/yyy):

The original documents are available at the site of the Spanish National Transplant Organization (ONT):
http://www.ont.es/infesp/Programa%20Marco%20de%20calidad%20y%20Seguridad/1.%201.%20Proceso%20de%20consentimiento%20a%20la%20donaci%C3%B3n%20de%20fallecido.pdf
