# Supplementary material for: The influence of the native lung on early outcomes and survival after single lung transplantation
Source: PLoS One. 2021 Apr 7;16(4):e0249758. doi: 10.1371/journal.pone.0249758 (PMC8026083; doi:10.1371/journal.pone.0249758)
Supplement: Attachment — Submitted filename: Response Letter to Reviewers.docx [file pone.0249758.s003.docx]

**RESPONSE TO REVIEWERS**

We would like to thank the reviewers of PLOS ONE for taking the time to review our manuscript entitled THE INFLUENCE OF THE NATIVE LUNG ON EARLY OUTCOMES AND SURVIVAL AFTER SINGLE LUNG TRANSPLANTATION. We appreciate the valuable and detailed comments provided by the reviewers.

We have made some corrections and clarifications in the manuscript after going over the reviewer’s comments.

*Reviewer #1: The manuscript entitled "THE INFLUENCE OF THE NATIVE LUNG ON EARLY OUTCOMES AND SURVIVALAFTER SINGLE LUNG TRANSPLANTATION" is well written and a potential topic of interest in the field of lung transplant. The research finding is not novel and there is no direct evidence how the native lung is a source of morbidity. As the author mentioned that this topic is a very controversial, however, due to the correct, appropriately and rigorous data analysis I will recommend this paper for acceptance, and the controversies related to this topic I will leave on the readers.*

RESPONSE: We thank the reviewer for his/her comments to our manuscript.

*Reviewer #2: Most of LT are performed as the last therapeutic option in end stage degenerative lung disease as COPD-EMPHYSEMA and fibrotic lung disease, mainly IPF. The last decade was marked by a clear shift to BLT vs. SLT due to surgical, prognostic (mortality/survival) causes. Although world statistics still present almost half of patients receiving SLT the BLT trend mainly in very active transplant centers is clear. Focusing on lung grafts as a source of lower survival in SLT is limited as the authors noticed. The native lung was analyzed by various groups before as to its impact on survival
The authors add a local retrospective study to the existing data and looked on the impact of the native lung status on survival following SLT. There are 2 main flaws in the methodology as far as I understand:*

*1. One may not decide the impact on survival on patients following SLT using any parameter without comparing to BLT. As mentioned by the authors this lack of comparison is a limitation, in my opinion a fundamental one. You may not recommend BLT for COPD patients instead of SLT without comparing relevant data from BOTH options.*

RESPONSE: We thank the comments of the reviewer. It is clear that we cannot recommend a bilateral procedure over a unilateral lung transplant on the basis of the analysis or single lung transplants alone. We agree with the reviewer that, for these purposes, the cohort of BLT should have been included in the study. However, the analysis of bilateral procedures was out of the focus of the present analysis. Our purpose has to know what happened with the native lung, irrespective of the behaviour of the lung graft, and whether the native complications could impact on early outcomes and survival. For this reason, according to the suggestions of the reviewer, we have eliminated recommendations of BLT for COPD instead of SLT because this statement is not supported by the data reported in our study.

*2. I am confused by comparing the survival and mortality data. All patients without native lung complications presented higher 30 days mortality than those with (even when divided between COPD and IPF although not significant). On the other hand survival was worse in COPD patients with native lung complications (both early and late) but not in IPF patients. There is no clear explanation to this aparent discrepancy both in text and figure legends.*

RESPONSE: We thank the reviewer comments. This is one of the major observations of our study: the fact that the native lung is not responsible of major complications leading to death in the early post-transplant period (30-days). Our analysis confirms that 30-day mortality after SLT occurs as a consequence of complications related to the lung graft (not the native), in addition to other well-known causes of early death (cardiac failure, infectious complications). On the contrary, long-term survival was adversely affected by complications in the native lungs, especially in COPD patients with lung cancer arising in the native lung. As shown in figure 1, only one case (IPF group) (2%) died due to native complications (native lung pneumonia), as opposed to 16 (15%) of early deaths that were unrelated to native lung problems. As only one case died in the early post-transplant period as a consequence of a native complication, differences of survival, especially for COPD, are related with late complications (rather than early complications), especially lung neoplasms in the native. This is more clearly demonstrated when analysing survival in the cohort of patients surviving 30 days post-transplant.

*Reviewer #3: The topic is of interest for the lung transplant community.
English language is the first main limitation of the manuscript. Many paragraphs are very hard to understand.*

RESPONSE: Thank you for your comments. An extensive review of English language has been done. English grammar, expressions, and syntax corrections have been made throughout the text. Some paragraphs have been shortened to make them more understandable.

*The design of the study is somehow confusing and should be clearly presented upfront. The reader is expecting to understand the object of your analysis, the outcome measures, and the methodology of your analysis.*

RESPONSE: Thank you for your comments. This is an observational analytic retrospective case-control study to determine the influence of native lung complications on early outcomes and survival after SLT for IPF or COPD. The primary end-points are 30-day mortality and survival. This clarification has been included in methods section (lines 86-96).

*The statistical methodology is also confusing and should be reviewed both for the descriptives and the regression analysis.*

RESPONSE: Thank you for your valuable comments. The description of the statistical methods was reviewed by our statistician without observing major flaws in the description. Nevertheless, changes have been included to better describe the statistics, according to the suggestions of the reviewer (lines 137-141). Also, we have included information regarding the availability of the data set used for the present analysis (lines 176-180).

*I also suggest to redraft your discussion and try to define the key results of the study. The discussion is very long and the take-home messages are not clearly presented. Please shorten your text and make it simple.*

RESPONSE: Thank you for you comments. We agree with the reviewer that the Discussion is too long. We have made extensive changes throughout the text to make it clearer. We believe that the main message is more clearly presented.

*Overall, I think that you should consider revising the structure and presentation of your study. The topic is interesting and deserves the work and time of a revision.*

RESPONSE: We wish to thank the reviewer for taking the time to review our manuscript.
